# Reply to Acerbo et al. Do Beta-Blockers Really Matter in Patients with Myocardial Infarction Without Left Ventricular Systolic Dysfunction? Comment on “Sabina et al. Beta-Blockers in Patients with Myocardial Infarction and Preserved Left Ventricular Ejection: A Systematic Review and Meta-Analysis of Randomized Controlled Trials. *J. Clin. Med.* 2025, *14*, 150”

**DOI:** 10.3390/jcm14072249

**Published:** 2025-03-26

**Authors:** Michael Sabina, Jennifer Trube, Graciela Luna, Anas Bizanti

**Affiliations:** Department of Internal Medicine Graduate Medical Education, Lakeland Regional Health Medical Center, Lakeland, FL 33805, USA; jennifer.trube@mylrh.org (J.T.); graciela.luna@mylrh.org (G.L.); anas.bizanti@mylrh.org (A.B.)

We appreciate the insightful commentary by Acerbo et al. [1] regarding our recent meta-analysis on the role of beta-blockers in patients with myocardial infarction (MI) and preserved left ventricular ejection fraction (LVEF). The discussion surrounding the use of beta-blockers in this population remains highly relevant, particularly in light of advancements in revascularization and medical therapy.

## Historical Context

Acerbo et al. emphasize the historical foundation of beta-blocker therapy in post-MI management, particularly from studies conducted in the pre-reperfusion era [2,3,4]. Beta-blockers have been integral in reducing mortality and morbidity in patients with heart failure and reduced ejection fraction (HFrEF). However, the contemporary management of MI has evolved significantly, with the widespread adoption of primary percutaneous coronary intervention (PCI), high-potency statins, dual antiplatelet therapy, and renin–angiotensin–aldosterone system (RAAS) inhibitors. Given these developments, it is essential to reassess whether beta-blockers offer additional benefit in MI patients with preserved ejection fraction.

Acerbo et al. highlight the differences in guideline recommendations regarding beta-blocker use. The American College of Cardiology (ACC) and American Heart Association (AHA) guidelines do not advocate for the routine use of beta-blockers in stable MI patients with preserved LVEF, whereas the European Society of Cardiology (ESC) provides a broader recommendation supporting beta-blockers in acute coronary syndrome. This discrepancy underscores the lack of definitive evidence and the need for further research to clarify the role of beta-blockers in this population.

## Clinical Relevance of Increased Heart Rate and Blood Pressure

The ABYSS trial reported an increase of 10 beats per minute in heart rate and a mild rise in systolic and diastolic blood pressure after beta-blocker discontinuation. However, these physiological changes are expected with withdrawal and do not necessarily indicate clinical harm. Without significant differences in mortality or cardiovascular outcomes, these changes are likely either transient or not meaningful in patients with preserved myocardial function. Additionally, if blood pressure control is a concern, the 2024 European Society of Cardiology (ESC) hypertension guidelines now classify beta-blockers as an alternative treatment to angiotensin-converting enzyme (ACE) inhibitors, angiotensin receptor blockers (ARBs), angiotensin receptor-neprilysin inhibitors (ARNIs), calcium channel blockers (CCBs), diuretics, and sodium-glucose cotransporter-2 (SGLT2) inhibitors [5].

## Potential Bias in Increased Hospitalization Rates

The ABYSS trial also noted an increase in hospitalizations for coronary-related reasons following beta-blocker discontinuation, including angina and ischemia leading to angiography [6]. However, hospitalization is often a subjective decision influenced by physician perception of risk, particularly when a patient is known to be off beta-blockers. Furthermore, patients aware of their beta-blocker discontinuation may have reported anginal symptoms more frequently due to psychological factors. Despite these hospitalizations, the trial did not show any differences in mortality or major cardiovascular events. Alternative therapies such as nitrates and calcium channel blockers remain viable first-line options for managing anginal symptoms and preventing unnecessary hospitalizations.

## Quality of Life and Potential Confounders

The commentary references the lack of quality-of-life improvement in the ABYSS trial using the EQ-5D questionnaire. However, this tool may not be optimal for assessing post-MI patients, as it focuses primarily on musculoskeletal health. Additionally, post-MI patients commonly have comorbidities such as diabetes, obesity, hypertension, and established CAD, all of which can independently impact quality of life. Depression is also prevalent in this population, with studies showing potential benefit from SSRIs in post-MI patients as seen in the Sertraline Antidepressant Heart Attack Randomized Trial (SADHART) and a meta analysis in 2020 which showed potential cardiovascular benefits with SSRIs in this patient population [7,8]. Given these confounders, it is difficult to attribute quality-of-life outcomes solely to beta-blocker discontinuation.

## Safety and Side Effect Profile

The REDUCE-AMI trial remains the only study to specifically assess the adverse effects of beta-blockers following MI [9]. While it was not adequately powered to detect rare side effects, no significant differences were found in hospitalization rates for bradycardia, advanced AV block, hypotension, syncope, pacemaker implantation, or the exacerbation of asthma or COPD. As noted in the commentary, safety remains a critical factor in evaluating the long-term role of beta-blockers. Ongoing studies will help clarify whether their discontinuation has any long-term safety implications.

## Third-Generation Beta-Blockers and Their Role

The CAPITAL-RCT trial exclusively studied carvedilol, a third-generation beta-blocker, yet it did not demonstrate superiority over placebo in preventing cardiovascular events [10]. Given that patients with preserved LVEF do not have significant structural impairment, the potential additional benefits of newer beta-blockers with vasodilatory and antioxidant properties may not be clinically meaningful in this group. However, this does not discount their well-documented advantages in patients with HFrEF.

## Beta-Blockers in Patients with Mid-Range Ejection Fraction (mrEF)

When considering MI patients with mid-range ejection fraction (LVEF 40–49%), uncertainty surrounding the benefit of beta-blockers is even greater. Beta-blockers may provide anti-ischemic and anti-remodeling effects in this subgroup, yet robust data remain lacking. The ABYSS trial included only 338 patients with mrEF, while the REDUCE-AMI trial did not enroll any patients from this category. The CAPITAL trial also lacks detailed subgroup analysis on these patients. This limitation underscores the need for further evidence to determine the role of beta-blockers in post-MI patients with mrEF.

As Acerbo et al. have correctly noted, the REBOOT-CNIC, BETAMI, DANBLOCK, and SMART-DECISION trials will address many unresolved questions raised by ABYSS, providing greater clarity on beta-blocker utility in MI patients without significant systolic dysfunction. These trials will allow for subgroup analyses, assessing whether beta-blockers provide differential benefits or potential harm in mrEF versus preserved EF populations. Furthermore, BETAMI and DANBLOCK will evaluate long-term safety outcomes, expanding on the findings from REDUCE-AMI. The large patient cohorts from these studies will likely enable strong evidence-based recommendations on beta-blocker use in contemporary post-MI care. Additionally, as Acerbo et al. mention, the SMART-DECISION trial (Long-term Beta-blocker Therapy After Acute Myocardial Infarction) is evaluating beta-blocker discontinuation in patients with stable coronary artery disease who are considered at low risk after their index event. As Acerbo et al. suggest, these studies will provide valuable insight into whether beta-blockers remain essential in this subset of patients or if they can be safely de-prescribed in patients with preserved LVEF [11,12,13,14].

## Future Directions for Beta-Blocker Therapy

A recent editorial published in *The New England Journal of Medicine* by Tomas Jernberg, MD, Ph.D., underscores the importance of waiting for the results of ongoing trials before definitively updating guidelines. He notes that ‘it is prudent to wait for the results of additional ongoing trials of beta-blockers involving patients with MI and a preserved left ventricular ejection fraction before definitively updating guidelines’ and that ‘the results of the ABYSS, REDUCE-AMI, and ongoing trials will most likely provide firm evidence regarding beta-blocker treatment in this patient population.’
